# Additive effects of type 2 diabetes and metabolic syndrome on left ventricular torsion and linear deformation abnormalities during dobutamine stress echocardiography

**DOI:** 10.3389/fcvm.2022.991415

**Published:** 2022-09-08

**Authors:** Falah Aboukhoudir, Clothilde Philouze, Antoine Grandperrin, Stéphane Nottin, Philippe Obert

**Affiliations:** ^1^UPR4278 LaPEC, Laboratory of Experimental Cardiovascular Physiology, Avignon University, Avignon, France; ^2^Cardiology Department, Duffaut Hospital Center, Avignon, France

**Keywords:** uncomplicated type 2 diabetes, metabolic syndrome, asymptomatic patients, dobutamine stress echocardiography, speckle-tracking imaging, twist-untwist mechanics

## Abstract

**Objective:**

The interplay between metabolic syndrome (MS) and type 2 diabetes (T2D) on regional myocardial mechanics and the potential additional effects of their combination remain poorly understood. In this context, we evaluated left ventricular (LV) torsion and linear deformation at rest and under dobutamine (DB) stress in patients with T2D, MS or both.

**Methods:**

Thirty-nine T2D patients without MS (T2D), 37 MS patients free from T2D (MS), 44 patients with both T2D and MS (T2D-MS group) and 38 healthy patients (control group) were prospectively recruited. Speckle-tracking echocardiography (STE) was conducted at rest and low dose DB to evaluate LV myocardial longitudinal (LS) as well as circumferential (CS) strain and early diastolic strain rate (LSrd, CSrd) and twist-untwist mechanics.

**Results:**

At rest, MS, T2D and controls presented with similar resting LS and LSrd while significant lower values were obtained in T2D-MS compared to controls. DB revealed reduced LS, LSrd, CS and CSrd in MS and T2D groups compared to controls. In T2-MS, the decline in LS and LSrd established at rest was exacerbated under DB. Stress echocardiography revealed also lower basal rotation and subsequently lower twist in MS and T2D patients compared to controls. T2D-MS showed major impairments of apical rotation and twist under DB stress, with values significantly lower compared to the 3 other groups. From stepwise multiple linear regression analysis, epicardial adipose tissue for Δ (rest to DB) LS, numbers of MS factors for Δ CS and Δ Twist emerged as major independent predictors.

**Conclusion:**

These results demonstrate synergic and additive effects of T2D and MS on LV torsion and linear deformation abnormalities in asymptomatic patients with metabolic diseases. They also highlight the usefulness of speckle tracking echocardiography under DB stress in detecting multidirectional myocardial mechanics impairments that can remain barely detectable at rest, such as in isolated T2D or MS patients.

## Introduction

Type 2 diabetes mellitus (T2D) is a major risk factor for cardiovascular diseases and is associated with the development of a specific cardiopathy, the diabetic cardiomyopathy (1). Diabetic cardiomyopathy is the leading cause of morbidity and mortality in T2D patients and is thought to develop rapidly after the onset of diabetes (2). Early diagnosis and cardiac follow-up of patients are therefore of paramount importance. In a previous study, we reported the usefulness of speckle-tracking echography under dobutamine (DB) stress in unmasking multidirectional early left ventricle (LV) dysfunction in asymptomatic T2D patients (3). Indeed, in this population, deformation indexes were similar to those of aged and sex-matched healthy subjects at rest. The latter finding is, however, not unanimously reported in the literature, some authors observing an altered regional myocardial function at rest (4–10), while others reported results consistent with ours (11, 12). Such inconsistencies may originate from variability in the clinical characteristics of the studied populations. Indeed, factors such as glycemic control or the presence of extracardiac complications and comorbidities can influence the extent of the cardiac alterations (13–20). Of note, the presence of concomitant metabolic syndrome (MS) could worsen the impairment of regional myocardial function. MS is frequent in T2D patients and is a well-recognized risk factor for cardiovascular diseases and heart failure (21, 22). Previous studies from our laboratory already highlighted major impairments of longitudinal strain (LS) in asymptomatic MS patients (23, 24), a finding in line with other investigations led in similar populations (25–27). Moreover, the components of MS (dyslipidemia and elevated abdominal adiposity, blood pressure and fasting glycemia) are individually associated with LV dysfunction, the cardiovascular risk increasing with each additional MS factor (23, 27). However, to our knowledge, no study has yet depicted the interplay between MS and T2D on regional myocardial function and the potential additional deleterious effect of their combination.

Most of the aforementioned studies focused on longitudinal deformations solely, while a knowledge of the multidirectional deformations is crucial for a better understanding of the extent of myocardial damages at a subclinical stage. Indeed, LS are mainly driven by the LV subendocardial layer, while the median and subepicardial layers mostly govern the circumferential strains (CS) and torsional mechanics. LS are therefore often impacted early, being sensitive to microvascular dysfunction, while CS and torsional mechanics are affected in more advanced stages (28). Yet, data regarding the multidirectional impact of MS on regional myocardial function are sparse and conflicting (23, 26, 27). Although the variability in the characteristics of the populations enrolled is likely to be a contributing factor, it must be underlined that these previous evaluations were carried out at rest. Similarly to what we previously reported in T2D patients, resting deformation imaging might not be sensitive enough to detect subtle dysfunctions, and the early alterations of myocardial regional mechanics may require the use of stress echocardiography to be unmasked (3). DB challenge could notably uncover a blunted response of myocardial torsional mechanics, the latter playing a central role in heart response to increased workload (29).

The aims of the present study were then to perform a comprehensive, multidirectional characterization of LV regional myocardial function at rest and under DB stress in asymptomatic T2D, MS and T2D-MS patients and to evaluate the potential synergic and additive effects of the MS and T2D combination compared to T2D and MS alone.

## Materials and methods

### Study population

**FIGURE 1:**
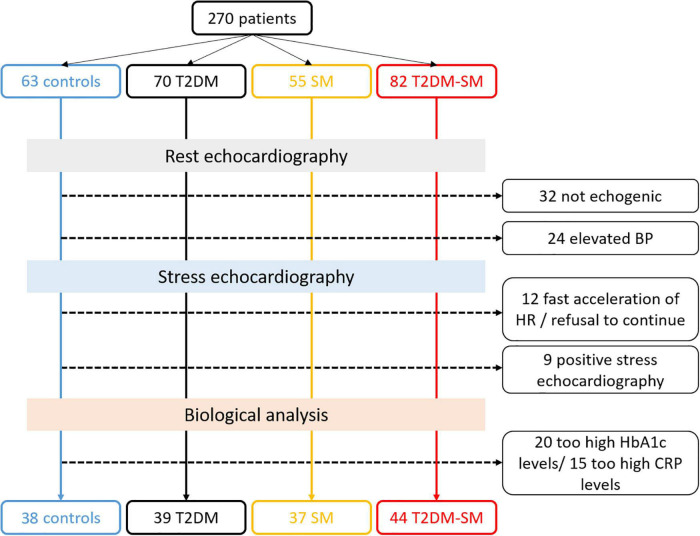
Flow chart of patient recruitment with exclusion criteria consideration.

According to the aims of this study, patients with T2D and/or MS (30) were prospectively recruited from our cardiology department from January 2015 to July 2020. The exclusion criteria were poor echogenicity, severe obesity, insulin therapy, LV ejection fraction < 55%, known cardiovascular diseases, and T2D-related complications, including moderate to severe autonomic neuropathy, proliferative retinopathy, and nephropathy. All the subjects were free from epicardial coronary disease, attested by negative findings on a high-dose of DB stress echocardiography (40 μg.kg^–1^.min^–1^) or coronary angiography when appropriate. Patients with hypertension but well-controlled blood pressure were included, those with superior to grade 1 hypertension were excluded. A total of 39 T2D patients without MS (T2D group), 37 MS patients free from T2D (MS group) and 44 patients with both T2D and MS (T2D-MS group) were enrolled. Thirty-eight healthy patients matched for sex, with similar age and normal echocardiographic findings, were also enrolled during routine checkups as a control group. As previously described (3), a medical survey was performed to check for exclusion/inclusion criteria and clinical data. Then, blood samples were collected in a fasting state for measurement of biological data such as glycemia, glycated hemoglobin, total cholesterol, high- and low-density lipoproteins (HDL, LDL), triglycerides, ultra-sensitive C-reactive protein or pro-brain natriuretic peptide. The study protocol was approved by the local human ethics committee (IRB-15/05.01) and all subjects provided written informed consents. The Figure 1 represents the flowchart of our study population.

### Echocardiography recordings

All patients underwent both resting and stress echocardiography, with the subject in left lateral decubitus position, using commercially available ultrasound equipment (Vivid E95; GE Healthcare, Milwaukee, WI, United States). At rest and each step of stress echocardiography protocol, bidimensional cine loops of the LV were recorded in parasternal short axis (base, papillary muscles and apex), parasternal long axis and apical 4- and 2-chamber views and saved for blinded offline analysis. Grayscale images were saved at a frame rate of 80–90 fps. Stress echocardiography was performed using the same protocol that was previously described by our group (3). Briefly, DB was infused intravenously in incremental doses of 10, 20, 30 and 40 μg.kg^–1^.min^–1^ in 3-min stages. The test was stopped when age-adjusted heart rate limit was reached, when severe hypertension (systolic BP > 250 mmHg or diastolic BP > 120 mmHg) or symptomatic reduction in systolic BP > 40 mmHg from baseline developed, or when ventricular arrhythmia occurred. β-blocker intake was interrupted at least 72 h prior to DB stress echocardiography.

### Echocardiography analysis

A blinded data analysis was performed post-processing by one experienced reader (EchoPAC 201; GE Healthcare) and all measurements were average from 3 cardiac cycles. Intra and inter-observer reliability at rest and during low-dose DB of deformation imaging indexes has been reported in detail elsewhere (3), with good concordance correlation coefficients (*r* > 0.82 in each case). All standard echocardiographic and Doppler parameters of LV systolic and diastolic function were measured according to recent guidelines (31). Speckle tracking echocardiography analysis was performed in accordance with guidelines of American Society of Echocardiography and European Association of Cardiovascular Imaging (31).

### Left ventricular morphology and global function at rest

Left ventricular end-diastolic (LVEDV) and end-systolic (LVESV) volumes and ejection fraction were measured using the Simpson biplane method. LV mass was calculated using the Devereux formula and indexed to height (Cornell adjustment). LV end-diastolic volumes were used as preload index. LV diastolic function was assessed from peak early (E wave) and atrial (A wave) transmitral flow velocities. Peak e’ average from septum and lateral walls and E/e’ ratio were evaluated using Tissue Doppler and used as indices of LV relaxation and filling pressures, respectively (32). Epicardial adipose tissue was identified and measured as previously described (33). To estimate cardiac afterload, the meridional wall stress was calculated according to the formula proposed by Jamal et al. (34).

### Left atrial morphology

According to current recommendations (35), left atrial volume (LAV) was calculated using the biplane method in apical 4- and 2-chamber views and indexed to body surface area.

### Left ventricular strain and twist mechanics

The 2D-strain analysis was conducted at rest and at a DB stage corresponding to a target heart rate between 110 and 120 bpm, in order to ensure sufficient temporal resolution for robust speckle tracking. For each view, the 3 cardiac cycles displaying the best image quality were selected. LS and CS and their corresponding early diastolic strain rate (LSrd and CSrd, respectively) as well as twist mechanics (apical and basal rotations, twist and twisting/untwisting rates) were obtained as previously detailed by our group (36). Briefly, EchoPAC data were exported as “.txt files” in order to be processed with a specific toolbox (Scilab version 4.1; Consortium Scilab, INRIA-ENPC, Paris, France). To adjust all strain parameters for inter-subject differences in heart and frame rates, the time sequence was also normalized to the percentage of systolic duration (i.e., time was 100% at end systole). Spreadsheet calculation allowed the detection of peak LSrd and CSrd, peak of LV twist and untwist rates, and the time to these peaks. A quality control was finally performed by an experienced investigator in order to replace or delete abnormal data. For each parameter, delta values (Δ) calculated as the difference between data at low dose DB minus data at rest were obtained.

### Statistical analysis

Statistical analysis was performed using IBM SPSS statistics 20.0™ software (IBM Corp., United States). Values are expressed as mean ± SD, otherwise specified. Statistical significance was defined as *p* value < 0.05. Normality was assessed using Shapiro–Wilk test and non-Gaussian biological variables were log-transformed. Biological, demographic variables and resting standard echocardiographic data were analyzed using a generalized linear model (GLM). Chi-square test was used for categorical data. Deformation imaging indexes were analyzed using a linear mixed-effect model with random intercept and group and conditions as well as age (when applicable, see Table 4) as fixed effects. In case of significant interaction between groups and conditions, a GLM was used for group’s comparison at rest and low-dose DB while a *t*-test for paired samples assessed DB response within each group. Covariates such as loading indexes, hypertension, medical treatment, inflammation, body mass index (BMI) or wait circumference were included in the different models, when appropriate. Inter-group comparison for delta (Δ) values was assessed using a GLM. Pearson correlations were used to investigate the association between LS, CS, apical rotation or Twist measured under DB stress condition as well as their Δ changes from rest to low dose DB with clinical, biological and echocardiographic parameters. A multiple stepwise linear regression analysis was used to determine the independent predictors of Δ values for each STE variable, with *p* < 0.15 in bivariate testing used as a requirement to enter the model.

**TABLE 1:** Clinical characteristics.

	Controls	T2D	MS	T2D-MS	*P* value
**Demographic characteristics**					
Gender *M/F*	19/19	25/14	20/17	29/15	0.40
Age (years)	51 ± 7	54 ± 9	52 ± 8	58 ± 6^***,†††,#^	< 0.001
Body mass index (kg/m^2^)	24.1 ± 3.6	26.1 ± 3.8^***^	29.2 ± 3.9^***,###^	28.7 ± 3.6^***,###^	< 0.001
Waist circumference (cm)	80 ± 15	95 ± 13^***^	104 ± 9^***,###^	104 ± 11^***,###^	< 0.001
Number of SM factors	0.9 ± 0.8	1.8 ± 0.4^***^	3.6 ± 0.7***,^###^	3.7 ± 0.7^***,###^	< 0.001
Increased BP (%)	8 (21)	15 (40)^ ***^	29 (78)^ ***,###^	38 (86)^ ***,###^	< 0.001
Increased plasma glucose (%)	2 (5)	39 (100)^ ***^	28 (75)^ ***,##^	44 (100)^ ***,††^	< 0.001
Increased abdominal obesity (%)	19 (50)	35 (89)^ ***^	35 (95)^ ***^	43 (98)^ ***^	< 0.001
Hypertriglyceridemia (%)	5 (13)	8 (20)	28 (75)^ ***,###^	25 (57)^ ***,##^	< 0.001
Decreased HDL (%)	25 (65)	29 (74)	28 (75)	38 (86)	0.18
Smoking *n (%)*	12 (31)	11 (28)	12 (32)	17 (38)	0.78
**Biological parameters**					
HDL cholesterol (g/L)	0.54 ± 0.11	0.50 ± 0.14	0.45 ± 0.11^**^	0.42 ± 0.10^***,##^	< 0.001
LDL cholesterol (g/L)	1.26 ± 0.28	1.17 ± 0.37	1.40 ± 0.36^##^	1.16 ± 0.45^††^	0.01
Triglycerides (g/L)**^§^**	1.10 ± 0.50	1.15 ± 0.34	2.07 ± 1.40^***,###^	1.62 ± 0.67^***,##^	< 0.001
Fasting glycemia *g/L*	0.89 ± 0.07	1.38 ± 0.57^***^	1.05 ± 0.11^*,###^	1.39 ± 0.32^***,†††^	< 0.001
HbA1c%	5.4 ± 0.3	7.5 ± 1.9^***^	5.6 ± 0.3^###^	7.4 ± 1.3^***,†††^	< 0.001
CRPus (g/L)**^§^**	1.2 ± 0.7	2.3 ± 2.0^**^	2.1 ± 1.8^**^	3.6 ± 3.7^***,†^	< 0.001
Pro-BNP (pg/mL)**^§^**	6.7 ± 2.5	9.1 ± 11.6	7.2 ± 6.6	7.2 ± 5.5	0.47
**Medications**					
Antihypertensive drugs *n (%)*	-	3 (8)	15 (40)^ ##^	24 (54)^ ###^	< 0.001
Antidyslipidemic drugs *n (%)*	-	10 (25)	12 (32)	19 (44)^ **^	0.88

M/F, males or females; BP, blood pressure; HDL and LDL, high- and low-density lipoproteins; HbA1c, glycated hemoglobin; CRPus, ultra-sensitive c-reactive protein; *Pro-BNP*, pro-brain natriuretic peptide.

*p < 0.05, **p < 0.01, ***p < 0.001 vs. controls; ^#^textitp < 0.05, ^##^*p* < 0.01, ^###^*p* < 0.001 vs. T2D; ^†^*p* < 0.05. ^††^*p* < 0.01, ^†††^*p* < 0.001 vs. MS; ^§^data were log transformed before statistical analysis.

**TABLE 2:** Echocardiographic characteristics.

	Controls	T2D	MS	T2D-MS	*P* value
**2D/TM**					
LAV (ml/m^2^)	22.6 ± 7.1	23.2 ± 6.3	22.5 ± 9.1	26.4 ± 8.6^*,†^	0.04
LVEDV (ml/m^2^) (ml/m^2^)	40.1 ± 6.5	38.9 ± 10.1	38.5 ± 8.9	39.9 ± 8.4	0.82
LVESV (ml/m^2^) (ml/m^2^)	14.5 ± 3.9	13.3 ± 4.4	13.2 ± 3.9	14.2 ± 4.4	0.45
IVSd (mm)	8.0 ± 1.5	7.9 ± 1.3	8.3 ± 1.6	9.7 ± 1.7^***,###,†††^	< 0.001
PWd (mm)	7.8 ± 1.3	7.6 ± 1.5	8.2 ± 1.7	9.2 ± 1.9^***,###,†^	< 0.001
RWT	0.32 ± 0.06	0.32 ± 0.05	0.33 ± 0.07	0.40 ± 0.10^***,###,†^	< 0.001
LVmass/height^2.7^ (g/m^2.7^)	32.1 ± 9.8	32.7 ± 8.2	34.7 ± 8.4	38.4 ± 9.2^**,##,†^	0.007
LVEF Simpson (%)	63.7 ± 7.7	65.2 ± 5.7	65.1 ± 7.7	63.4 ± 7.3	0.52
EAT (mm)	3.2 ± 1.1	5.2 ± 1.7^***^	5.2 ± 1.6^***^	6.8 ± 1.9^***,###,†††^	< 0.001
**Pulsed Doppler**					
E (cm/s)	77.1 ± 16.4	74.2 ± 14.8	77.6 ± 15.4	79.0 ± 15.8	0.56
E/A	1.04 ± 0.33	0.99 ± 0.24	1.05 ± 0.29	0.94 ± 0.22	0.20
**TDI parameters**					
s’ (cm/s)	9.2 ± 2.0	8.5 ± 2.2	8.2 ± 1.4	8.2 ± 1.9	0.12
e’ (cm/s)	10.3 ± 2.7	8.5 ± 1.8^***^	9.2 ± 1.9^**^	8.3 ± 1.9^***,†^	< 0.001
E/e’ ratio	7.8 ± 1.9	8.8 ± 1.7*	8.7 ± 1.8*	10.0 ± 2.9^***,#,††^	< 0.001

*LAV*, left atrial volume indexed; *LV-ED/-ESV*, end-systolic/end-diastolic left ventricle volume indexed to body surface area; *IVSd*, end-diastolic interventricular septum thickness; *PWd*, end-diastolic posterior wall thickness; RWT, relative wall thickness; *LVEF*, left ventricle ejection fraction; *EAT*, epicardial adipose tissue; *E*, mitral flux early diastolic wave; *A*, mitral flux late diastolic wave; s′, systolic mitral annulus tissue velocity; e′, early diastolic mitral annulus tissue velocity.

*p < 0.05, **p < 0.01, ***p < 0.001 vs. controls; ^#^textitp < 0.05, ^##^*p* < 0.01, ^###^*p* < 0.001 vs. T2D; ^†^*p* < 0.05. ^††^*p* < 0.01, ^†††^*p* < 0.001 vs. MS.

**TABLE 3:** Hemodynamic and speckle tracking echocardiography data.

	Rest				GLM	DB				GLM	MM
	Controls	T2D	MS	T2D-MS	_*P*_	Controls	T2D	MS	T2D-MS	_*P*_	_*p*_
**STE Deformation imaging**											
LS (%)	−21.3 ± 1.7	−20.4 ± 2.6	−20.6 ± 3.5	−19.5 ± 2.7^**^	0.03	−24.3 ± 2.5	−22.3 ± 2.4^**^	−22.1 ± 3.1^***^	−20.6 ± 2.3^***,##,††^	< 0.001	T:<0.001 G: < 0.001 T*G:0.13
LSrd (s^–1^)	1.4 ± 0.4	1.3 ± 0.4	1.4 ± 0.5	1.1 ± 0.3^**,†^	0.04	1.9 ± 0.4	1.6 ± 0.4^**^	1.7 ± 0.4*	1.4 ± 0.4^***,#,†^	< 0.001	T:<0.001 G: < 0.001 T*G:0.31
CS (%)	−21.6 ± 3.1	−20.0 ± 3.3	−20.6 ± 3.4	−21.2 ± 3.7	0.26	−27.0 ± 4.0	−23.7 ± 4.1^**^	−24.9 ± 4.8*	−23.7 ± 4.5^**^	0.008	T: < 0.001 G:0.006 T*G:0.16
CSrd (s^–1^)	1.8 ± 0.6	1.4 ± 0.4^**^	1.5 ± 0.5*	1.5 ± 0.4^**^	0.02	2.5 ± 0.8	2.0 ± 0.6^**^	2.1 ± 0.7*	1.8 ± 0.5^***,†^	< 0.001	T:<0.001 G: < 0.001 T*G:0.13
Basal Rotation (°)	−7.6 ± 2.7^‡‡‡^	−6.9 ± 3.5^‡‡‡^	−7.1 ± 3.6^‡‡‡^	−7.7 ± 3.0^‡‡‡^	0.63	−14.0 ± 4.6	−11.5 ± 4.5^**^	−10.1 ± 3.0^***^	−11.5 ± 3.5^**^	0.001	T: < 0.001 G:0.007 T*G:0.04
Apical rotation (°)	9.5 ± 4.3^‡‡‡^	12.4 ± 4.2^**,‡‡^	12.6 ± 5.5^**,‡^	11.6 ± 5.3*	0.03	16.3 ± 7.4	15.2 ± 5.9	14.6 ± 8.0	10.8 ± 4.0^***,##,††^	0.01	T: < 0.001 G:0.02 T*G: < 0.001
Twist (°)	16.0 ± 6.2^‡‡‡^	18.0 ± 4.9^‡‡‡^	18.4 ± 6.4^‡‡^	18.2 ± 7.5^‡^	0.66	29.2 ± 8.4	25.3 ± 8.1*	22.5 ± 9.8^***^	20.6 ± 6.0^***,##^	< 0.001	T:<0.001 G:0.04 T*G:0.01
Twisting rate (°/s)	102 ± 39^‡‡‡^	109 ± 32^‡‡‡^	116 ± 38^‡‡‡^	112 ± 49^‡‡‡^	0.56	255 ± 68	232 ± 57	209 ± 61^**,#^	200 ± 51^***,#^	< 0.001	T:<0.001 G:0.03 T*G: < 0.001
Untwisting rate (°/s)	−122 ± 56	−117 ± 48	−121 ± 45	−102 ± 45	0.32	−250 ± 82	−239 ± 82	−208 ± 63^**^	−190 ± 55^***,##^	0.003	T: < 0.001 G:0.001 T*G:0.09
**Hemodynamics**											
HR (bpm)	74 ± 8	75 ± 9	73 ± 9	74 ± 10	−	112 ± 7	110 ± 6	111 ± 7	110 ± 4	−	T: < 0.001 G:0.75 T*G:0.49
SBP (mmHg)	124 ± 12	124 ± 13	132 ± 13^**,##^	130 ± 11*	0.04	138 ± 11	137 ± 13	136 ± 12	141 ± 17	0.40	T: < 0.001 G:0.04 T*G:0.11
DBP (mmHg)	70 ± 10	71 ± 10	76 ± 18	−75 ± 11	−	72 ± 10	75 ± 9	73 ± 13	72 ± 11	−	T: < 0.98 G:0.18 T*G:0.12
σ _*es*_ (g/cm^2^)	81.2 ± 31.1	72.6 ± 22.2	82.3 ± 22.1	75.6 ± 20.9	0.37	51.2 ± 25.7	45.3 ± 15.8	58.6 ± 19.3^##^	43.5 ± 14.8^†††^	0.02	T: < 0.001 G: < 0.009 T*G:0.73
EDV (ml)	71.7 ± 15.4	78.0 ± 21.8	75.4 ± 18.9	78.0 ± 18.6	−	66.6 ± 17.2	74.4 ± 18.2	72.6 ± 21.2	73.4 ± 19.2	−	T: < 0.01 G:0.15 T*G:0.12

*HR*, heart rate; *SBP/DBP*, systolic/diastolic blood pressure; *LS*, longitudinal strain; *LSrd*, early diastolic longitudinal strain rate; *CS*, circumferential strain; *CSrd*, early diastolic circumferential strain rate; σ_*es*_, end-systolic meridional wall stress; *EDV*, end-diastolic volume.

*p < 0.05, **p < 0.01, ***p < 0.001 vs. controls; ^#^*p* < 0.05, ^##^*p* < 0.01, ^###^*p* < 0.001 vs. T2D; ^†^*p* < 0.05. ^††^*p* < 0.01, ^†††^*p* < 0.001 vs. MS; ^‡^*p* < 0.05, ^‡‡^*p* < 0.01, ^‡‡‡^*p* < 0.001 vs. DB in the same group.

**TABLE 4:** Univariate and multivariate correlation analysis of delta changes in STE indexes with clinical, biological and echocardiographic indexes.

	Dobutamine								Univariate							
	LS		CS		Apical rot.		Twist		Δ LS		Δ CS		Δ Apical rot.		Δ Twist	
	r	p	r	p	r	p	r	p	r	p	r	p	r	p	r	p
Gender	–0.15	0.06	–0.02	0.86	**0.17**	**0.03**	0.14	0.09	–0.05	0.52	0.10	0.26	**0.16**	**0.04**	0.07	0.39
Age	**0.28**	**0.001**	0.09	0.27	–0.12	0.13	–0.06	0.45	**0.22**	**0.007**	**0.21**	**0.01**	−**0.19**	**0.02**	–0.15	0.09
Hypertension	**0.26**	**0.001**	0.07	0.37	−**0.19**	**0.02**	−**0.24**	**0.005**	**0.17**	**0.03**	**0.18**	**0.03**	−**0.19**	**0.02**	−**0.20**	**0.01**
Smoking	–0.01	0.85	–0.07	0.36	−**0.17**	**0.03**	−**0.20**	**0.01**	0.02	0.74	–0.05	0.57	–0.07	0.37	–0.08	0.31
MS number	**0.41**	*bf* < 0.001	**0.21**	**0.01**	−**0.19**	**0.01**	−**0.29**	**0.001**	**0.22**	**0.007**	**0.26**	**0.003**	−**0.31**	*bf* < 0.001	−**0.34**	*bf* < 0.001
BMI	**0.17**	**0.03**	**0.18**	**0.03**	0.13	0.11	−**0.23**	**0.007**	**0.16**	**0.04**	**0.18**	**0.04**	−**0.25**	**0.003**	−**0.29**	**0.001**
WC	**0.27**	**0.001**	**0.27**	**0.001**	–0.07	0.33	−**0.19**	**0.02**	**0.16**	**0.04**	**0.18**	**0.03**	−**0.23**	**0.008**	−**0.27**	**0.01**
HDL	–0.13	0.10	−**0.18**	**0.04**	0.12	0.16	**0.18**	**0.03**	0.001	0.98	–0.003	0.98	**0.19**	**0.02**	**0.19**	**0.02**
TG^§^	0.15	0.07	**0.18**	**0.04**	0.06	0.43	–0.01	0.84	0.07	0.34	0.04	0.63	–0.03	0.66	0.05	0.54
CRPus^§^	**0.17**	**0.03**	**0.22**	**0.01**	–0.05	0.53	–0.06	0.42	0.08	0.33	**0.19**	**0.03**	−**0.19**	**0.03**	−**0.19**	**0.03**
HbA1c	**0.26**	**0.001**	**0.18**	**0.04**	**0.17**	**0.04**	–0.10	0.21	0.06	0.42	0.08	0.50	−**0.23**	**0.008**	−**0.15**	**0.04**
Glycemia	**0.25**	**0.007**	**0.19**	**0.02**	**0.16**	**0.04**	–0.11	0.18	0.06	0.41	0.10	0.23	−**0.18**	**0.02**	–0.08	0.36
T2DM dur.	0.03	0.77	–0.03	0.79	–0.18	0.10	–0.02	0.84	–0.14	0.21	–0.02	0.86	–0.18	0.13	−**0**.08	0.46
RWT	**0.28**	**0.001**	–0.02	0.77	−**0.25**	**0.002**	−**0.26**	**0.002**	0.05	0.54	–0.01	0.89	–0.10	0.22	–0.05	0.52
LVMi	**0.19**	**0.01**	–0.04	0.64	−**0.16**	**0.04**	−**0.21**	**0.01**	–0.01	0.90	–0.03	0.68	–0.11	0.19	–0.12	0.16
EDV	0.006	0.43	0.16	0.06	–0.02	0.79	–0.13	0.12	0.05	0.49	–0.07	0.42	0.13	0.23	–0.05	0.57
σ_*es*_	–0.07	0.38	0.02	0.81	0.08	0.32	–0.008	0.92	–0.05	0.54	–0.03	0.67	0.09	0.29	0.09	0.30
EAT	**0.60**	*bf* < 0.0001	**0.18**	**0.04**	−**0.28**	**0.001**	−**0.33**	*bf* < 0.001	**0.36**	*bf* < 0.0001	**0.19**	**0.04**	−**0.28**	**0.001**	−**0.26**	**0.004**

Δ, delta value; *LS*, longitudinal strain; *CS*, circumferential strain; rot., rotation; *BMI*, body mass index; *WC*, waist circumference; HDL, high-density lipoproteins; TG, triglycerides; *CRPus*, ultra-sensitive c-reactive protein; *HOMA-IR*, homeostasis model assessment of insulin resistance; *dur*., duration; ΔSI-DI, strain imaging diastolic index obtained from apical views for ΔLS, and from short-axis views at apex for Δapical CS and rot, at papillary muscles for Δmid CS and at base for Δbasal rot; LVMi, left ventricular mass indexed to height; EAT, epicardial adipose tissue thickness; §, data were log transformed before statistical analysis. Bold values indicate significant results.

## Results

### Baseline clinical and echocardiographic characteristics

The clinical data are presented in Table 1. Populations were matched on sex, but T2D-MS were slightly older than the 3 other groups. Body mass index and waist circumference were significantly higher in the 3 groups of patients when compared to controls, with values being also significantly greater in MS and T2D-MS than T2D. Similar results were obtained regarding the number of MS factors. Additionally, among the 5 MS components, no inter-group differences were noticed for the prevalence of low HDL while significant differences were obtained in each case in the patients compared to controls for the prevalence of the 4 other variables. The 2 groups with MS presented with also a greater prevalence of increased BP and hypertriglyceridemia compared to T2D and, as expected, the 2 groups with T2D had a higher prevalence of increased plasma glucose compared to MS. Concerning the lipid profile, no differences were noticed between controls and T2D patients. The 2 groups with MS had reduced HDL and increased triglycerides compared to controls. Triglycerides were especially elevated in MS, values being also greater than in the 2 other diabetic groups. As expected, fasting glycemia and glycated hemoglobin were greater in T2D and T2D-MS than in MS and controls, while no differences were obtained between the 2 diabetic groups. Fasting glycemia was also slightly increased in MS compared to controls. Noteworthy, ultra-sensitive C-reactive protein levels were also substantially increased in the 3 groups of patients compared to controls, with values being also higher in T2D-MS compared to MS.

Results of conventional echocardiography are summarized in Table 2. LAV indexed was significantly increased in T2D-MS patients compared to MS and controls. While no inter-group differences were noticed for LVEDV and LVESV indexed, values of LV wall thicknesses as well as relative wall thickness and LV mass indexed were significantly higher in the T2D-MS patients compared to the 3 other groups. Similar values of LV ejection fraction, E and E/A ratio were noticed between the 4 groups. No inter-group differences were obtained for longitudinal systolic velocities by TDI while early diastolic velocities and E/e’ ratio were significantly decreased and increased, respectively, in the 3 groups of patients compared to controls. Of note, differences were also significant in T2D-MS compared to MS. Epicardial adipose tissue was markedly increased in T2D-MS patients compared to the 3 other groups, with values being also higher in MS and T2D patients than in controls.

### Speckle-tracking echocardiography at rest and under dobutamine infusion

**FIGURE 2:**
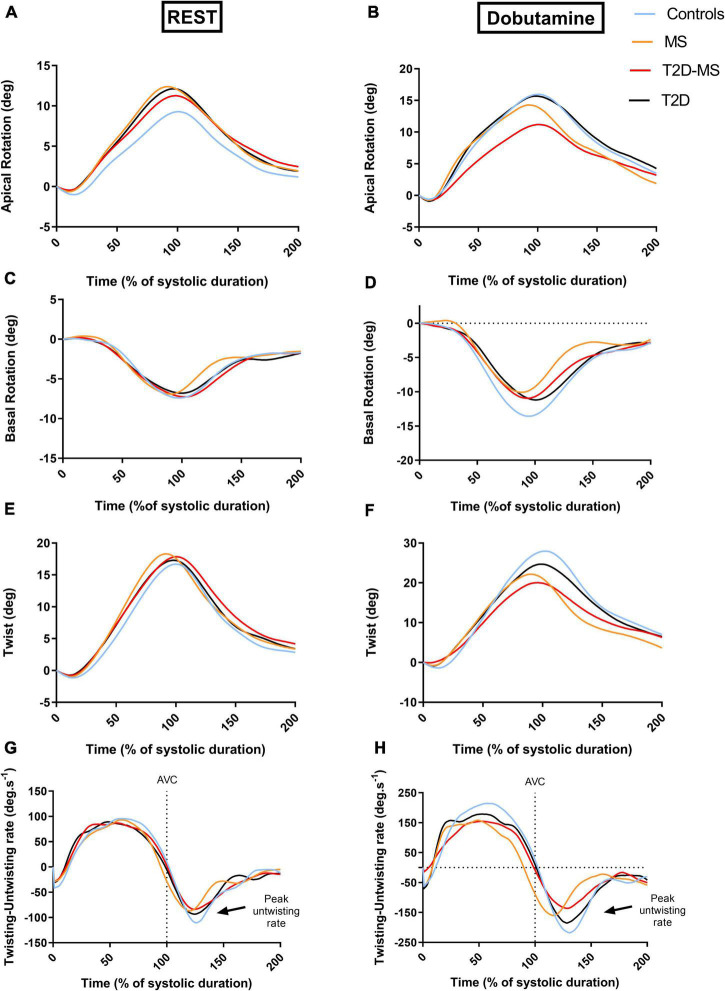
Patterns of apical **(A,B)** and basal rotations **(C,D)** as well as twist and twisting/untwisting rates **(E,F)** all over the cardiac cycle at rest **(A,C,E,G)** and low dose of dobutamine **(B,D,F,H)**. The curve for each group was obtained from averaging data of all the subjects. For each group, data of the curves were averaged at every percent of systolic duration from data of all subjects.

Speckle tracking echocardiography data are presented in Table 3 and Figure 2. At rest, no inter-group differences were noticed for twist mechanics (e.g., twist as well as twist and untwist rates), basal rotation and Cs. The 3 groups of patients presented with, however, greater apical rotations than controls. While no differences were obtained between MS, T2D and controls for LS and LSrd, values in T2D-MS patients were significantly reduced compared to controls. Low-dose DB revealed major inter-group differences for most speckle tracking echocardiography indexes. Indeed, LS, LSrd, CS and CSrd were now significantly decreased in MS and T2D compared to controls. Additionally, the reductions of LS and LSrd observed at rest in T2D-MS were further exacerbated under DB, differences being significant not only compared to controls but also to MS and T2D. As for MS and T2D, Cs and CSrd were also significantly reduced in T2D-MS compared to controls. While no differences were obtained under DB between MS, T2D and controls for apical rotation, lower basal rotation and subsequently twist were demonstrated in these 2 groups of patients compared to controls. Apical rotation and twist were, however, dramatically impaired in T2D-MS patients, with values reduced significantly compared to controls, but also to T2D and MS for apical rotation and in T2D only for twist. Overall, twist mechanics was predominantly depressed in the 2 groups presented with MS. All speckle tracking echocardiography indexes were significantly increased in the 4 groups in response to DB infusion, except for apical rotations that did not change in T2D-MS patients.

Results of the univariate analysis of speckle tracking echocardiography indexes under low dose DB conditions as well as in response to low dose DB (e.g., Δ changes from rest to low dose DB) are depicted in Table 4. For most of the indexes, good correlations were obtained with age, number of MS factors, hypertension, metabolic markers of T2D, abdominal obesity, inflammation and epicardial adipose tissue. From stepwise multiple linear regression analyses, epicardial adipose tissue (β = 0.34, *p* < 0.001; r^2^ = 0.12, *p* < 0.001) for Δ SL, numbers of MS factors for Δ CS and Δ Twist (β = 0.27, *p* = 0.003; r^2^ = 0.08, *p* = 0.003 and β = −0.30, *p* < 0.001; r^2^ = 0.09, *p* < 0.001) and number of MS factors and presence of T2D (β = −0.27, 0.002; β = −0.18, 0.03, r^2^ = 0.13, *p* < 0.001) for Δ apical rotation emerged as major independent predictors. The inter-group differences under DB stress previously mentioned for LS, apical and basal rotations as well as twist were still evident after accounting for, in addition to age and sex, others potential confounding factors such as hypertension, abdominal obesity and systemic inflammation. For CS, differences between controls and the 3 other groups under DB disappeared when waist circumference and ultra-sensitive C-reactive protein were introduced as covariates. For twist, differences between patients and controls under DB were still evident when hypertension and abdominal obesity were used as covariates, but disappeared between T2D and T2D-MS. All speckle tracking echocardiography variables were, however, not influenced by medical treatments. Indexes of loading conditions did not differ between the 4 groups at rest. They were, however, significantly impacted by DB. However, no correlations were demonstrated between loading conditions and speckle tracking echocardiography indexes under stress conditions.

## Discussion

To our knowledge, the present study is the first one to comprehensively assess regional myocardial mechanics under DB stress in asymptomatic T2D, MS and T2D-MS patients and to evaluate the potential synergic and additive effect of the T2D-MS combination compared to T2D and MS alone. The major findings were, firstly, that patients with combined T2D and MS exhibited globally a more important impairment of regional myocardial function compared to patients with only T2D or MS and, secondly, that only DB stress allowed unmasking alterations that were otherwise undetectable at rest in these last 2 groups of patients.

The present findings further illustrate the challenges in the characterization of cardiac impairments in patients with metabolic diseases and the pitfalls of comparing studies with different patient inclusion criteria and clinical characteristics in this area. Indeed, most studies focusing on cardiac regional function in metabolic disease did not discriminate between patients with T2D or MS only and patients with combined T2D-MS, while factors associated with T2D, such as glycemic control or MS components may influence the extent of cardiac dysfunction (13, 18–20, 23, 25). As a matter of fact, in our study, only patients with combined T2D-MS exhibited at rest an altered longitudinal function, with significantly reduced LS and LSrd, while patients with isolated T2D or MS presented with a preserved longitudinal function. This observation could help explaining, at least in part, the discrepancies found in the literature regarding the presence or not of a decreased LS in patients with T2D (4–12).

While literature is extensive on longitudinal function in metabolic disorders, comparative data are scarcer regarding circumferential and rotational parameters, despite their key role in cardiac performance. Indeed, the LV twist motion induced by apical counterclockwise and basal clockwise rotations in systole aids ventricular ejection, while early diastolic untwist generates suction and facilitates diastolic filling (37). Systolic twist acts to limit myocardial energy expenditure by creating high intraventricular systolic pressures with minimal muscle shortening, resulting in efficient LV contraction (37). Moreover, the resultant elastic recoil of the LV has important implications for diastolic filling. In fact, the elastic recoil occurring during early diastole is thought to be a result of the vigorous contraction and compression of cardiac proteins such as titin (38). The potential energy stored in the spring like titin is unleashed during diastole, promoting myocardial relaxation and diastolic filling (38). In our study, basal rotations, twist and untwisting rate were preserved at rest, whereas apical rotations were significantly enhanced in patients (e.g., T2D, MS or both) compared to controls. These salient findings differed from those previously reported. Indeed, Tadic et al. (27) showed an increase in both twist and untwisting rate at rest in MS patients. Interestingly, in this paper, authors described a progressive increment of twist-untwist parameters with each additional MS factor (from 1 to 5). Another study reported preserved rotations and twist in patients with MS, and an increase of untwisting rate (23). Tadic et al. (8) and Crendal et al. (23) also reported an increase of twist and untwisting rate in T2D patients compared to controls. However, in these studies, the authors did not distinguish between isolated MS and T2D, which may have probably impacted their results. In our study, the increase in apical rotation probably acts as a compensatory mechanism to preserve twist, and so ejection, in patients with T2D and/or MS.

Although our results indicate an overall preservation of cardiac function in patients with isolated T2D or MS, the absence of myocardial dysfunction cannot be ascertained, as an evaluation at rest may not be sensitive enough in these populations. Indeed, as we previously demonstrated in T2D patients, an adrenergic stress may reveal functional impairments that remained otherwise subtle at rest (3). However, in this previous work, we did not discriminate between T2D and T2D-MS patients. The present results further specify the myocardial regional function impairments that DB stress echocardiography allows unmasking in these different groups of patients with metabolic diseases. To our knowledge, we are the first to carry out such an investigation. Our results revealed interesting specificities between groups. Indeed, patients with T2D showed an alteration of basal rotation and twist, while patients with MS presented an additional alteration of untwisting rate, and patients with both T2D-MS exhibited severe alterations in all these components of myocardial mechanics, with an additional drop of apical rotations. These salient findings revealed a continuum from T2D or MS alone to combination of both T2D and MS and strengthen the importance of stress echocardiography, especially in asymptomatic T2D and MS patients for whom impairments remain discreet at rest. The lack of functional reserve in rotational mechanics we unveiled in T2D-MS is of paramount importance because it plays a major role in myocardial performance under stress. Indeed, in such conditions (e.g., physical exercise or dobutamine infusion), the elevated heart rate results in reduced diastolic filling time, requiring the diastolic function to be subsequently drastically improved to attain the same end-diastolic volume in a shorter amount of time (38). In athletes, previous studies have shown the importance of untwisting rate in facilitating LV filling (29, 39, 40). These results might thus help understanding, at least in part, the development of severe exercise intolerance previously reported in diabetic populations (41, 42). Furthermore, the drop in torsional deformations may profoundly affect LV function by altering the distribution of LV wall stresses and therefore myocardial strains. Previous studies have indeed suggested that torsion may serve to equalize transmural sarcomere shortening, and so reduce transmural gradients of oxygen utilization, wall stress, and contractile work during ejection (43). A reduction in torsional deformations could thus increase the gradient of sarcomere work and oxygen utilization across the myocardium, accelerating the development of subendocardial fibrosis (44). In line with this hypothesis, Zhang et al. (45) reported an association between LV twist and myocardial fibrosis in patients with hypertrophic cardiomyopathy. These alterations could lead to a lower cardiomyocyte ability to store elastic energy and may explain, consequently, the drop of untwisting rate. Taken together, our results underline the importance of characterizing LV twist-untwist adaptations in patients with T2D, MS or both, for a better understanding of exercise intolerance mechanisms in each population and the implementation of relevant interventions.

Even if this study was not intended to investigate the mechanisms underlying cardiac function impairments in our patients, our regression analyses offered some interesting leads. As could have been expected, MS factors and T2D parameters were global contributors to the blunted response to DB stress. However, these purely metabolic variables were statistically overwhelmed by epicardial adipose tissue thickness, that showed strong correlations with all deformation indexes. In previous studies from our laboratory, we already observed close relationships between epicardial adipose tissue thickness and regional myocardial function impairments in T2D or MS populations (3, 24). This fat pad has gained growing attention over the last years as its expansion has been associated with cardiovascular damages, such as arterial stiffness and inflammation, hemodynamic impairments and myocardial injury-related biomarkers, in metabolic diseases or heart failure (46–50). Indeed, while being cardioprotective in a physiological context, this adipose tissue switches to a deleterious, pro-inflammatory secretory profile in a pathological environment (51, 52). As a matter of fact, *in vitro* studies have reported that exposing cardiomyocytes to the epicardial adipose tissue secretome from T2D patients induces an alteration of calcium fluxes, insulin resistance and contractile dysfunction (53). Epicardial adipose tissue is thus know being recognized as a valuable target in cardiometabolic diseases (50) and our results tend to support that epicardial adipose tissue implication should be considered and investigated at the earliest stages of myocardial dysfunction.

### Clinical implications

The detection of early signs of myocardial alteration is of paramount importance to improve patient care by preventing the development of irreversible damages and the progression to heart failure. The present study emphasizes that rest echocardiography is not sufficient enough to ascertain the absence of nascent cardiac dysfunction in asymptomatic patients with metabolic diseases and underlines the usefulness of performing a stress echocardiography in unmasking early myocardial function impairments, especially in case of isolated MS or TD2. Our results also emphasize that asymptomatic patients with T2D or MS only or a combination of T2D-MS do not present with the same alterations of LV myocardial mechanics.

## Study limitations

The main limitation of this study is the choice to perform the analyses under low dose DB, at submaximal heart rate. This decision was taken to minimize the impact of DB on loading conditions, but also to ensure a sufficient image quality for speckle-tracking analysis (54). In future studies, it would, however, be of interest to conduct the assessments at maximal heart rate, under physiological stress conditions such as physical exercise, provided that the inherent image quality limitations can be overcome. This would have the additional advantage of allowing a better understanding of the myocardial mechanisms of exercise intolerance in these different populations.

The second limitation to this study is that echocardiographic epicardial adipose tissue thickness measurement does not take into account the 3D shape and volume of this fat pad and is therefore no gold standard for its evaluation. However, Iacobellis et al. (33) found a good correlation with MRI volume measurements, and the reproducibility of epicardial adipose tissue measurements is good in our laboratory (24).

### Conclusion

Collectively, our results clearly demonstrate the usefulness of speckle tracking echocardiography under DB stress in detecting multidirectional myocardial mechanics impairments that can remain barely detectable at rest in asymptomatic patients with metabolic diseases. These results also highlight the different levels of impairment according to the patient’s clinical profile. This should be kept in mind when setting up a preventive cardiac check-up for patients with metabolic disorders.

## Data availability statement

The raw data supporting the conclusions of this article will be made available by the authors, without undue reservation.

## Ethics statement

The studies involving human participants were reviewed and approved by the local human ethics committee (IRB-15/05.01). The patients/participants provided their written informed consent to participate in this study.

## Author contributions

FA contributed to conception of the project, submission to the ethics committee, participants recruitment, echocardiographic recordings, writing of the manuscript, and final approval of the manuscript. PO and CP contributed to conception of the project, submission to the ethics committee, echocardiographic data analysis, statistical analysis, writing of the manuscript, and final approval of the manuscript. AG contributed to echocardiographic data analysis, statistical analysis, writing of the final manuscript, and final approval of the manuscript. SN contributed to conception of the project, echocardiographic data analysis, writing of the manuscript, and final approval of the manuscript. All authors are in agreement for the order of presentation of the authors and have read and approved the final version of the manuscript.
